# A systematic review of economic evaluations of CHW interventions aimed at improving child health outcomes

**DOI:** 10.1186/s12960-017-0192-5

**Published:** 2017-02-28

**Authors:** L. Nkonki, A. Tugendhaft, K. Hofman

**Affiliations:** 1 Centre for Health Systems and Services Revision, Division of Community Health, Faculty of Medicine and Health Sciences, Francie van Zijl Rylaan/Drive, Tygerberg, 7505 South Africa; 2 PRICELESS SA, MRC/Wits Rural Public Health and Health Transitions Research Unit (Agincourt), School of Public Health, Faculty of Health Sciences, University of the Witwatersrand, Johannesburg, South Africa

**Keywords:** Community health worker, Economic evaluations, Child health, Cost-effectiveness

## Abstract

Evidence of the cost-effectiveness of community health worker interventions is pertinent for decision-makers and programme planners who are turning to community services in order to strengthen health systems in the context of the momentum generated by strategies to support universal health care, the post-2015 Sustainable Development Goal agenda.

We conducted a systematic review of published economic evaluation studies of community health worker interventions aimed at improving child health outcomes. Four public health and economic evaluation databases were searched for studies that met the inclusion criteria: National Health Service Economic Evaluation Database (NHS EED), Cochrane, Paediatric Economic Evaluation Database (PEED), and PubMed. The search strategy was tailored to each database.

The 19 studies that met the inclusion criteria were conducted in either high income countries (HIC), low- income countries (LIC) and/or middle-income countries (MIC). The economic evaluations covered a wide range of interventions. Studies were grouped together by intended outcome or objective of each study. The data varied in quality. We found evidence of cost-effectiveness of community health worker (CHW) interventions in reducing malaria and asthma, decreasing mortality of neonates and children, improving maternal health, increasing exclusive breastfeeding and improving malnutrition, and positively impacting physical health and psychomotor development amongst children.

Studies measured varied outcomes, due to the heterogeneous nature of studies included; a meta-analysis was not conducted. Outcomes included disease- or condition -specific outcomes, morbidity, mortality, and generic measures (e.g. disability-adjusted life years (DALYs)). Nonetheless, all 19 interventions were found to be either cost-effective or highly cost-effective at a threshold specific to their respective countries.

There is a growing body of economic evaluation literature on cost-effectiveness of CHW interventions. However, this is largely for small scale and vertical programmes. There is a need for economic evaluations of larger and integrated CHW programmes in order to achieve the post-2015 Sustainable Development Goal agenda so that appropriate resources can be allocated to this subset of human resources for health. This is the first systematic review to assess the cost-effectiveness of community health workers in delivering child health interventions.

**Electronic supplementary material:**

The online version of this article (doi:10.1186/s12960-017-0192-5) contains supplementary material, which is available to authorized users.

## Background

Community services are seen as key for strengthening health systems in the context of the momentum generated by strategies for universal access to health care and the post-2015 Sustainable Development Goal agenda. Thus, the question of effectiveness and cost-effectiveness of community health worker (CHW) interventions is pertinent for decision-makers and programme planners. The choice of which CHW service or services to deliver needs to be underpinned by evidence of both effectiveness and cost-effectiveness. A substantial body of evidence on CHW intervention effectiveness exists using varied methodologies, time frames, and scope [1–7], but all six [1–6] reviews consistently highlight the scarcity of economic evaluations.

Evidence on costs and cost-effectiveness of CHW intervention is crucial for decision-making. CHW interventions are expected to have favourable economic evaluations because they are perceived to be cheap, fast, and easy. Largely because training of CHWs is significantly shorter than training of other health professionals (e.g. medical doctors and nurses), they extend coverage to geographically hard-to-reach populations and they are often paid a stipend or work as volunteers. In spite of all these factors, CHW programmes in many countries in the 1970s and 1980s were abandoned as they failed to realize the potential demonstrated in several initiatives led by nongovernmental organizations and in national programmes such as China’s “barefoot doctors”.

Several reviews [1–7] have reported that CHWs undertake a wide variety of tasks in primary and public health [4, 5] and CHWs deliver interventions in primary health care including nutrition, maternal and child health, malaria control, tuberculosis (TB) control, HIV/AIDS prevention and control, mental health, and non-communicable disease. A Cochrane review of CHW interventions identified 107 randomized control trials (RCTs) which showed promising benefits, compared to usual care, in increasing immunization uptake in children, improving breastfeeding rates until 6 months, reducing neonatal mortality, and improving pulmonary TB care rates. The review also reported that CHWs reduce child morbidity and child mortality, maternal mortality, and increase the likelihood of seeking care for childhood illness [8].

In 2009, a systematic review of economic evaluations of CHWs delivering vaccination programmes found only three studies that matched the inclusion criteria [9]. More recently, a non-systematic review on effectiveness identified nine specific areas in which CHWs are cost-effective: specific nutrition intervention, community-based therapeutic care for children with severe acute malnutrition, pneumonia control, diarrhoea prevention or treatment, malaria control, perinatal/neonatal care programmes, HIV/AIDS control in children, child survival programmes, and comprehensive primary health care with a community-based component [10]. Because this was not a systematic review, there is potential bias introduced during the search, selection of the studies, and interpretation of studies stages.

Systematic reviews aim to reduce bias in the estimation of the effects of a policy option by identifying all relevant studies, selecting those that meet explicit criteria, appraising their quality, and synthesizing the results using a transparent process. To address the current gap in knowledge regarding cost-effectiveness of CHW services for reducing child and maternal mortality, we have conducted a systematic review of economic evaluations of published economic evaluation studies aimed at improving child health outcomes.

## Methodology

### Search methods for identification of studies

We searched the following public health and economic evaluation databases for studies conducted globally on community health worker (CHW) interventions to improve child health outcomes: National Health Service Economic Evaluation Database (NHS EED), Cochrane, Paediatric Economic Evaluation Database (PEED), and PubMed. The search strategy was tailored to each database.

The functional definition of CHW was used, that being a member of the community who has received some training to promote health care or who carries out some health care services, but is not a professional. That definition covers a diverse cadre of workers, and at least 10 terms were used during searches as indicated in Additional file 1.

A combination of search terms was used in each of the databases, and these are shown in Additional file 1. We also searched for specific interventions such as breastfeeding, sanitation, hand-washing, immunization, and kangaroo mother care. We searched for studies conducted between 1980 and 2014.

#### Study eligibility

The key criterion for inclusion was that the study intervention included CHWs and an economic evaluation of the intervention was performed, with no publication restriction date. The review included full economic evaluations as they provide information on the relative trade-offs between the effect of the intervention on costs and outcomes and hence the most relevant information for health care decision-making [11]. Economic evaluations include cost-minimisation analysis (CMA)^1^, cost-effectiveness analysis (CEA)^2^, cost-utility analysis (CUA)^3^, cost-benefit analysis (CBA)^4^, and cost consequence (CC)^5^. All economic evaluations have two components; the first component is a costing analysis^6^ and the second component measures effects. All the above-mentioned economic evaluation studies (CMA, CEA, CUA, CBA, and CC) measure costs identically, but they differ in how they measure effects.

We excluded studies that (1) were published in languages other than English; (2) were not original studies; (3) did not provide any costing detail; (4) were designed such that CHWs were just one of several interventions being compared and it was difficult to distinguish the effect and costs of the CHW intervention.

#### Study selection

Two reviewers, with experience in health economics, were involved in the review process. One reviewer independently assessed the potential relevance of all titles and abstracts. Full-text copies of the articles identified as potentially eligible for inclusion were retrieved by the first reviewer. Assessment of the eligibility of interventions can vary between reviewers. Therefore, each full paper was evaluated independently for inclusion by both reviewers. Disagreements on the full-text articles were resolved through discussion between whichever two review authors the article was assigned to and, where necessary, by consulting a third author from the core team for an independent assessment. The final reviewing and writing of the summary and conclusions were done by the second reviewer.

### Data extraction

Two reviewers independently and in duplicate extracted data from each included study. A standard form was developed to extract descriptive and outcome. The form was based on a set of questions developed by Pegurri et al. [12] and Corluka et al. [9]. We have also added other questions based on our experience of CHW interventions such as retention of CHWs. The form’s appropriateness was assessed by piloting the form on selected full economic evaluation articles. The information that was extracted included:

1. Study area and population.
2. Perspective of the economic evaluation in other words was the economic evaluation conducted from a societal, provider, or patient perspective.
3. Timing of the economic evaluation.
4. Type of intervention delivered by CHW.
5. CHW role.
6. Training.
7. CHW retention (number of CHWs who have ceased to work as CHW in the project).
8. Incentives (financial and non-financial).
9. Duration of the intervention.
10. Study-type economic evaluation (e.g. is it a cost-effectiveness, cost analysis).
11. Comparator(s).
12. Costing approach.
  - Type of costs collected: financial or economic costs.
13. Outputs (unit costs).
14. Scale-up/operational scenarios (costs of replication/expansion).
15. Measure of effectiveness, e.g. as described by the authors of the reviews.
16. Economic evaluation outcomes (cost-effectiveness or cost utility analysis).
17. Funder.

It was not feasible to contact study authors to obtain any missing information.

## Results

### Description of studies

**Fig. 1:**
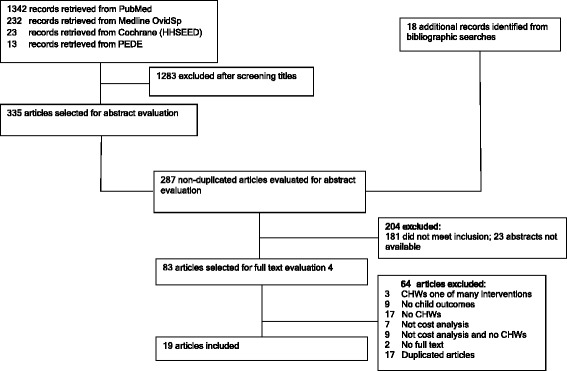
Flowchart showing the search, selection, and inclusion of studies

A total of 1610 titles and abstracts, written in English, were identified as shown in Fig. 1. Two hundred eighty-seven articles (excluding duplicates) were selected for abstract evaluation. An additional 18 records were identified from bibliographic searches. Eighty-three articles were considered potentially eligible for inclusion and full-text articles were obtained; 19 of these met the inclusion criteria (Tables 1 and 2).

**Table 1:** Economic evaluation studies by region

Developed countries	Developing countries	Developing countries
	Out of sub-Saharan Africa	Within sub-Saharan Africa
Morrell et al. 2006 (United Kingdom)	Gowani et al. 2014 [21] (Pakistan)	Tozan, 2010 [14] (Tanzania)
Pugh et al. 2002 [29] (USA)	Barzgar et al. 1997 [20] (Pakistan)	Gonzalez, 2000 [13] (Tanzania)
Frick et al. 2012 [28] (USA)	Hafeez et al. 2011 [22] (Pakistan)	Conteh et al. 2010 [15] (Ghana)
Margellos-Anast, 2012 [30] (USA)	Borghi, 2005 [23] (Nepal)	Nonvignon et al. 2013 (Ghana)
	Puett et al. 2013 [24] (Bangladesh)	Desmond et al. 2008 [17] (South Africa)
	San Sebastian et al. 2001 [25] (Ecuador)	Pagnoni et al. 1997 [18] (Burkina Faso)
	Aracena et al. 2009 [26] (Chile)	Chola, 2011 [19] (Uganda)
	Melville et al. 1995 (Jamaica) [27]

**Table 2:** Characteristics of included studies

Study	Setting	Cost year	Size of the population served by the programme being analysed	Currency	Type of evaluation	Method	Perspective	Target population/goal of intervention
Tozan, 2010 [14]	Broadly stated as rural African settings in which care seeking was low	2008	Cohort of 1 000 new born babies until 5 years of age	International dollar	Cost-effectiveness analysis	Decision tree used to model costs and impacts of treating severe childhood malaria with pre-referral artesunate. What would be the added gains if CHWs rather than health professionals are used?	Provider/health system	Children in rural areas where burden of malaria remains high
								*Malaria reduction*
Gonzalez, 2000 [13]	Tanzania (SSA)	1996	2 322 infants under 1 year	US$	Decision analysis/cost-effectiveness analysis	Used life table method to estimate number of years of life lost that would be prevented if 3 strategies (2 involving CHWs) were used to manage malaria and anaemia in children	Health provider/societal	Infants living in Kilombero district in 1996
								*Malaria/anaemia reduction*
Conteh et al. 2010 [15]	Hohoe district, Ghana	2008	1 801 children aged 2–59 months	US$	Cost-effectiveness analysis	Measured the costs and impacts of delivering malaria prophylaxis using CHWs (termed community-based volunteers) and how that differs with usual care and no intervention approach	Provider/societal	Children aged 3–59 months who resided in the study district
								*Malaria reduction*
Nonvignon et al. 2013	Rural Ghana	2009	13 135 children under the age of five	US$	Cost-effectiveness analysis based on cluster randomized trial	Compared the costs and impacts of using community health workers to manage fevers at home with standard practice of self-medication or seeking care at health centres	Societal	Febrile children under 5 years
								*Reducing mortality from suspected malaria/infections*
Pagnoni et al. 1997 [18]	Rural Burkina Faso	1994	35 000 mothers	US$	Cost-consequence analysis	Measured the costs and benefits of using community-based workers to provide prompt and adequate treatment for malaria and compared outcomes with pre-intervention period	Provider	Mothers within study setting
								*Reduce severity of malaria morbidity*
Chola, 2011 [19]	Uganda (SSA)	2007	406 breastfeeding mothers	US$	Cost analysis	Estimated actual costs incurred as a result of individual peer-counselling visits to breastfeeding mothers. Alternative peer support intervention modelled and cost	Local provider’s perspective	Pregnant women within the study sites
								*Promote exclusive breastfeeding*
Desmond et al. 2008 [17]	South Africa	N/A	2 781 pregnant women	US$	Cost-effectiveness analysis based on cohort study of pregnant women attending government antenatal clinic coupled with modelled analysis of alternative intervention	Compared the rates of exclusive breastfeeding when intervention was offered at different coverage levels	Health systems/provider	Pregnant women attending a government antenatal clinic
								*Promote exclusive breastfeeding*
Frick et al. 2012 [28]	Mid-Atlantic region, USA	N/A	328 low-income women	US$	Cost analysis	Measured the costs of providing support to breastfeeding low-income women and compared the costs offset as a result of reduced health care utilization	Provider	Women undergoing postpartum hospitalization at a large medical centre
								*Promote breastfeeding*
Pugh et al. 2002 [29]	Mid-Atlantic region, USA	N/A	41 low-income women	US$	Cost-effectiveness analysis	Compared cost-effectiveness of community-based randomized trial aimed at improving exclusive breastfeeding rates amongst low-income mothers against usual care	Societal	Women undergoing postpartum hospitalization at a large medical centre
								*Promote exclusive breastfeeding*
Morrell et al. 2006	United Kingdom		311 women	British Pound	Cost analysis	Cost and impact assessment of CHW providing postnatal support at home	Societal	Women delivering at Sheffield Hospital older than 17 years
								*Mainly maternal health outcomes plus breastfeeding*
Margellos-Anast 2012 [30]	USA	Not specified	135 women with children	US$	Cost analysis	Calculated costs of urgent health resource utilization averted in absence of intervention Urgent HRU = emergency visits, hospitalizations, and urgent clinic visits	Not specified	Asthmatic children within study setting of Chicago *Asthma*
Puett et al. 2013 [24]	Southern Bangladesh	2010	724 care givers	US$	Cost analysis	Compared the impact and costs of using CHWs to manage cases of malnutrition vs. facility-based inpatient treatment of SAM at health centres as the existing standard of care in Bangladesh	Societal	Children with severe acute malnutrition (SAM) in Bhola District
								*Malnutrition*
Melville et al. 1995 [27]	Jamaica	N/A	88 children	US$	Cost analysis	Measured nutritional status and growth of children whose caregivers received nutritional advice from CHWs pre-intervention and post-intervention	Provider	Children <36 months
								*Nutritional status*
Gowani et al. 2014 [21]	Rural Sindh, Pakistan	N/A	1 121 infants	US$	Cost-effectiveness analysis	Measured the improvement in cognitive, language, and motor development skills when responsive stimulation and enhanced nutrition were added into an existing package of services offered by lay health workers	Provider	Children less than 2 years
								*Improve early childhood development*
Aracena et al. 2009 [26]	Chile	N/A	45 adolescent	US$	Cost-effectiveness analysis	Compared what the rate of maternal depression and linguistic skills development of children would be when CHWs (termed health educators) provided home support to adolescent mothers vs. usual care at health facility	Not explicitly stated	Adolescent mothers
								*Children’s linguistic skills*
Barzgar et al. 1997 [20]	Rural Pakistan	N/A	Services provided to about 50 000 people	US$	Cost analysis	Measured crude birth rates, maternal mortality rates, and infant mortality rates following an intervention that utilized community health workers for promoting uptake of health services and family planning. Rates compared with pre-intervention period	Provider	Community within the 3 districts but primary focus seemed to be on women and children
								*Reduce under-5 mortality*
Hafeez et al. 2011 [22]	Pakistan	N/A	Each lay health worker served a population of 1 000 people. The programme employed 90 000 lay health workers.	US$	Cost analysis	Measured the reduction in mortality that resulted from using lay health workers to perform preventive activities and basic curative functions within the study site	Provider	Pregnant women, children under 5 years, couples in catchment population eligible to use contraception, general community
								*Improve maternal and child key health indicators*
Borghi, 2005 [23]	Rural Nepal (Asia)	2003	14 884—number of married women of reproductive age in the intervention area	US$	Cost-effectiveness analysis	Women’s groups as lay health workers—what would be the pregnancy outcomes if they did not exist? Based on cluster randomized trial	Provider	Women residing within the study population
								*Reduce neonatal mortality*
San Sebastian et al. 2001 [25]	Ecuador	1994	180 children less than 1 year old	US$	Cost-consequence analysis	Measured the costs and health impacts of using 2 different approaches to improve immunization, one using CHWs, another using health facility-level staff	Provider and patient	Children eligible for immunization
								*Immunization*

### Setting

The majority of economic evaluation studies took place in LMICs. Seven studies were conducted in sub-Saharan Africa: two in Tanzania [13, 14], two in Ghana [15, 16], one in South Africa [17], one in Burkina Faso [18], and one in Uganda [19]. Five studies were undertaken in Asia: three in Pakistan [20–22], one in Nepal [23], and one in Bangladesh [24]. Three studies were done LAC (Latin America and the Caribbean): one in Ecuador [25], one in Chile [26], and one in Jamaica [27]. Four of the 19 studies were performed in HIC countries: three in the United States of America, two of which were targeted to low-income populations [28–30], and one was done in the United Kingdom [31].

### Cost-effectiveness of the interventions

Economic evaluations of CHW interventions aimed at improving child health outcomes cover a wide range of interventions. Hence, we grouped together studies by intended outcome or objective of each study, as listed below. The three remaining studies were extremely diverse and could not be usefully grouped. Sixteen out of the 19 included studies were targeted at a specific outcome, and the remaining three had several health outcome goals.

Economic evaluations can be conducted alongside a primary epidemiological study, for instance alongside a randomized controlled trial or cohort study. The source of effectiveness data for an economic evaluation is not limited to only one primary study. Effectiveness data can also be collected from synthesizing published effectiveness studies [32]. The economic evaluations reviewed below included primary evaluations and piggyback studies, as well as studies based on previously published literature of costs and effectiveness data (Table 3).

**Table 3:** Economic evaluation results of included studies

Study	Costs measured	Measure of effectiveness or benefit	Economic results	Author conclusions
Tozan, 2010 [14]	Direct medical	Deaths DALYs averted	Low intervention uptake—low referral compliance scenario averts 1 death, 19 DALYs, at incremental cost of I$ 17 466, cost per DALY averted = 1 173 Full intervention uptake—full referral compliance scenario averts 37 deaths, 967 DALYs, at incremental cost of I$7 1 166, cost per DALY averted = 77	Pre-referral artesunate is cost-effective in rural African settings when referral compliance and intervention uptake are moderate or higher The intervention was cost-effective under all scenarios.
Gonzalez, 2000 [13]	Direct medical Direct non-medical Indirect costs	Years of life lost DALYs	All three intervention strategies cost-effective. Deltaprim administered by VHWs twice as effective as a combination of Deltaprim + iron administered by mothers	Results favour inclusion of malaria chemoprophylaxis and iron supplementation delivered through EPI
Conteh et al. 2010 [15]	Direct medical Direct intervention Indirect/productivity losses	Rebound in malaria morbidity Malaria cases averted	Intervention cost-effectiveness of the 2 drug therapies artesunate plus amodiaquine (AS + AQ) and sulphadoxine-pyrimethamine ranged from $61/malaria case averted (societal perspective) to $65/malaria case averted (provider perspective). Cost per child enrolled fell considerably when modelled to district level as compared to those encountered under trial conditions	Potential for the different treatment approaches to be cost-effective at district level when implemented by CHWs
Nonvignon et al. 2013	Direct Indirect costs	Malaria cases averted Deaths averted DALYs averted	Cost-effectiveness was better when CHWs managed fevers with antimalarial only without antibiotics. Compared to control arm : Each malaria case averted cost US$ 150.18 (antimalarial) and US$ 227.49 (antimalarial + antibiotic) Each death averted cost US$ 2 585.58 (antimalarial) and US$ 3 272.20 (antimalarial + antibiotic). Each DALY cost US$ 90.25 (antimalarial) and US$ 114.21 (antimalarial + antibiotic)	Home management of under-5 fevers by CHWs in rural settings is cost-effective in reducing under-5 mortality and cost less than the WHO threshold of $150/DALY averted
Pagnoni et al. 1997 [18]	Direct programme costs	Primary: proportional reduction in severe malaria cases Secondary: proportion of women who self-diagnosed malaria and sought “medical treatment” care from CHWs pre- and post-intervention period	Slight decrease in severe malaria cases reported Proportion of women who sought help from CHWs increased from 5 to 76% Proportion of mothers using modern tablets to treat malaria in children almost doubled Appropriate treatment increased from 3 to 49% Adequacy of length of treatment increased from 21 to 72% Costs: average net cost per resident child = US$ 0.06	Low-cost community-based intervention aimed at providing children with prompt and adequate treatment for malaria is possible Intervention could reduce morbidity of severe malaria
Chola et al. 2011 [19]	Direct costs	N/A	Total project costs = US$ 56 308; 53% of costs attributed to peer supervision (38% of costs due to transport); 26% attributed to peer support Alternative community EBF programme = $14 per visit; $74 per mother Cost of scaling up programme on population of 1 million = $2 590 000 Total cost per individual counselling visit = $26 Total cost per mother counselled = $139 Costs of alternative modelled intervention 80% lower	Costs of $139 per mother considered expensive. High costs driven by personnel salaries
Desmond et al. 2008 [17]	Direct costs	Months of EBF	Incremental costs per month of EBF associated with moving from the less effective scenarios to the more effective scenarios: Nothing—basic R616 ($88) Basic—simplified R162 ($23) Simplified—Full R879 ($126)	Modelled scenarios indicate that there is a possibility that costs and outcomes may differ in real-life setting The simplified scenario, with a combination of clinic and home visits, is the most efficient in terms of cost per increased month of EBF and has the lowest incremental cost-effectiveness ratio.
Frick et al. 2012 [28]	Partial direct costs (transport, personnel time)	Number of visits to a clinic Number of formula feedings Number of prescription medicines taken	The cost of the personnel and travel required for the intervention was $296 per woman. Health care use savings were significant for clinic visits at 4 weeks with intervention group expensing 40% less clinic visits	Support for breastfeeding by community health nurses and peer counsellors is partially offset by reducing medical care utilization and formula feeding costs
Pugh et al. 2002 [29]	Direct costs Indirect costs	Primary outcome: EBF rates at 3, 6 months; Secondary: frequency of illness	At 3 months: 45% EBF in intervention arm versus 25% in usual care; at 6 months, 30% EBF in intervention arm versus 15% in usual care. Infants in intervention group had fewer sick visits; intervention cost $301/mother. No incremental cost-effectiveness ratios	CHWs can increase BF duration and reduce costs especially costs of support
Morrell et al. 2006	Direct medical costs	Primary outcome: general health perception at 6 weeks. Secondary outcomes: mean Edinburgh Postnatal Depression Scale (EPDS), Duke Functional Social Support (DUFSS) scores, and breastfeeding rates	No significant differences in health outcomes between intervention and control group. The total mean NHS cost to 6-month follow-up for the intervention group was £180 per woman greater than for the control group (confidence interval, £79.60, £272.40).	Added cost of intervention at no benefit made intervention unfavourable though the service was valued by women
Margellos-Anast 2012 [30]	Direct medical costs	Asthma-related quality of life and number of urgent medical visits averted	Intervention saves US$ 2 561/participant, i.e. for every $1 spent on intervention, you save $5 Urgent HRU decreased from median of 4 to 1 (75% decline) Activity-limited days reduced from median of 7 to 3.5 (50% decline) Regular clinic visits increased from 2.5 to 3.5	CHW model is effective in improving asthma management Intervention decreased the need to use emergency health services by 75% CHW model improves asthma knowledge, quality of life Cultural competence key to success of intervention
Puett et al. 2013 [24]	Direct medical; direct non-medical; indirect costs	DALYS averted Deaths averted Child recovered Child treated	The community-based strategy cost US$ 26/DALY averted, compared with US$ 1 344 per DALY averted for inpatient treatment. The average cost to participant households for their child to recover from SAM in community treatment was one-sixth that of inpatient treatment	Community-based management of acute malnutrition (CMAM) delivered by community health workers (CHWs) is a cost-effective strategy compared with inpatient treatment and compares well with the cost-effectiveness of other common child survival interventions. In this context, inpatient treatment performed poorly in comparison with community treatment; even if performance was improved by 20%, it would remain over eight times less cost-effective than the CMAM intervention.
Melville et al. 1995 [27]	Direct costs	Percentage of children who gained adequate weight between May 1990 and Apr 1992	Cost per child of intervention = US$ 31.1 (annual cost of US$ 14.50); personnel comprised 75% of costs Malnutrition declined by 34.5%	CHVs can play a vital role in primary health care settings in developing countries
Gowani et al. 2014 [21]	Direct programme costs	Primary: cognitive, language, and motor development scores as measured by the BSID III criterion	Statistically significant improvements in primary outcome measures reported at 12 and 24 months when responsive stimulation (RS) was integrated into package but no additive benefits with RS + enhanced nutrition. CER ranged from $15–$19 per year (CER = annualized cost per LHW divided by composite scores of language, cognitive, or motor skills development)	With further refinement, integrating early stimulation with nutrition support can be scaled up effectively; on the basis of existing data in other settings, the cost-benefit to the country could be very significant.
Aracena et al. 2009 [26]	Direct medical costs	Linguistic skills development Nutritional state of mother Mental health of mother	Only the following showed statistically significant differences: (1) development of children’s language skills, (2) nutritional state of the mother, and (3) mental health of the mother. Cost of standard care = US$ 50 per adolescent over a period of 15 months, median cost of intervention for the home visit programme was US$ 90 per adolescent over a period of 15 months; total incremental cost of the home visit programme versus standard care US$ 40 per day over the same time period, i.e. the standard programme costs US$ 3.30 per month per adolescent, whilst the home visit programme costs US$ 6.	Intervention more effective at improving maternal outcomes.
Barzgar et al. 1997 [20]	Direct medical and non-medical	Proportional reduction in infant mortality, maternal mortality, increase in vaccination coverage	Costs per person = $0.39 for capital costs and $1.13 per person for recurrent costs. 50% reduction in infant mortality (from 130/1 000 at baseline to 64/1 000 after intervention) >50% reduction in maternal mortality—(596 per 100 000 at base line to 246 per 100 000 after intervention) >50% reduction in infant diarrheal deaths >97% reduction in neonatal tetanus No impact on low birth weight/malnutrition and pneumonia Immunization coverage increased by 80% in Chakwal, 70% Mastung and 100% in Malir	Capital and recurrent costs per person were lower than the allocations for public sector outlay in the same period of $1.87
Hafeez et al. 2011 [22]	Direct intervention costs	Key maternal and child health indicators: Contraceptive prevalence Fully immunized children Skilled birth attendance	CHW programme versus national averages: Fully immunized children—80 vs 47% IMR—51 vs 39% MMR—180 vs 276 Costs: average cost of each CHW = US$ 570 per year; salary costs comprised 50% of total	Focused mainly on impact rather than costs—CHWs effective in reducing MMR and improving vaccination coverage in rural areas. CHWs provide an important link between community and first level care. No detailed costs analysis reported—makes it difficult to judge cost-effectiveness of programme
Borghi, 2005 [23]	Direct costs	Neonatal mortality rate Life years saved (LYS)	Average annual cost per woman of reproductive age = $4.38 ($5.22 with health service strengthening) Average annual cost per newborn infant = US$ 22.51 ($26.82 with health systems strengthening (HSS)) Cost per neonatal death averted = $5 801 ($6 912 with HSS) Cost per life year saved = 211 (251 with HSS)	Intervention most suited to settings like Nepal where supply-side interventions may not be feasible due to resource requirements Personnel costs account for largest costs—70% Intervention likely to be more CE when replicated elsewhere due to lower start-up costs Intervention more CE when maternal LYS are included
San Sebastian et al. 2001 [25]	Direct intervention Indirect costs/productivity losses	Proportional increase in fully vaccinated children	Existent (Department of Health) strategy—$3 888 versus $3 618 for CHWs (no significance tests) Coverage of DPT3/polio was 22.6-fold higher in CHW strategy vs DH strategy	CHW strategy dominates the existent strategy Costs of averting disease not calculated which would have presented a stronger case for CHW model

#### Economic evaluations of CHW interventions aimed at reducing malaria in children

##### Setting

Five [13–16, 18] studies were conducted in sub-Saharan Africa. Two studies were conducted from a provider’s perspective [14, 18] and three from a societal perspective [13, 15, 16].

##### Description of interventions

The interventions were aimed at both community diagnosis and treatment of malaria. Malaria treatment included treatment for febrile and non-febrile children, many from a single dose of rectal antibiotics to 3 days of medication bimonthly.

The cost-effectiveness of the interventions was measured using generic economic evaluation measures, mortality, disease- or condition-specific measures, and intervention process measures. Three studies [13, 14, 16] measured cost-effectiveness using disability-adjusted life years (DALYs). DALYs combine years of life lost because of premature death with years of life lived with disability in one outcome measure. One study used years of life lost (YLL) [13]. Two studies measured deaths averted [14, 16]. Other measures of cost-effectiveness included intervention uptake [14, 18], referral compliance [14], anaemia [13], malaria cases averted [15, 16], adequacy of length of treatment [18], and appropriate treatment [18]. All studies aimed at reducing malaria in children were found to be cost-effective.

##### Results

The intervention of one dose of rectal artesunate by a CHW to a child with suspected severe malaria alongside referral advice to caregivers was found to be cost-effective [14]. The intervention was estimated to avert 19 DALYs (95% CI 16–21) at a cost of $1173 per DALY averted when the uptake and compliance were both at 25%. When the uptake and compliance were both 100%, the intervention could avert 967 DALYs (95% CI 884–1050) at a cost of $77 per DALY averted.

The three intervention strategies (Deltaprim and iron, Deltaprim, iron) for the prevention of severe anaemia and malaria in infants were found to be cost-effective compared to standard case management [13]. For the prevention of severe anaemia and from the perspective of the health provider, the cost-effectiveness ratios were, respectively, US$ 8, US$ 9, and US$ 21 per DALY for malaria chemoprophylaxis with Deltaprim and iron, Deltaprim alone, and iron alone. For malaria prevention, Deltaprim and iron cost US$ 9.7 per DALY and Deltaprim alone cost US$ 10.2 per DALY. From a sociocultural perspective, the cost-effectiveness ratios ranged from US$ 9 to US$ 26 for severe anaemia prevention and US$ 11 to US$ 12 for the prevention of clinical malaria.

The two strategies of home management of under-five fevers in Ghana using antimalarial-only artesunate-amodiaquine (AAQ) and combined treatment using antimalarial and antibiotics AAQ and artesunate-amodiaquine-amoxicillin (AMX) [16]. The cost per anaemia case averted was US$ 150.18 for AAQ and US$ 227.49 for AAQ and AMX, and the cost per death averted was US$ 2585.58 for AAQ and US$ 3272 for AAQ + AMX. Cost per DALY averted were US$ 90.25 for AAQ and US$ 114.21 for AAQ and AMX.

Intermittent preventive treatment for malaria in children (IPTc) was shown to be cost-effective [15]. During the intervention period, artesunate (AS) and amodiaquine (AQ) monthly was the most cost-effective IPTc drug regimen at US$ 67.77 (61.71–74.75, 95% CI) per malaria case averted based on intervention costs only, US$ 64.93 (58.92–71.92, 95% CI) per malaria case averted once the provider cost savings are included, and US$ 61.00 (54.98, 67.99, 95% CI) when direct household cost savings are also taken into account. Sulphadoxine-pyrimethamine (SP) bimonthly was US$ 105.35 (75.01–157.31, 95% CI), and AS and AQ bimonthly was US$ 211.80 (127.05–399.14, 95% CI) per malaria case averted based on intervention costs only. The incidence of malaria in the post-intervention period was higher in children who were less than 1 year old when they received AS and AQ monthly compared to the placebo group leading to higher cost-effectiveness ratios when 1-year follow-up is included.

A community-based programme to provide prompt and adequate treatment of presumptive malaria in children was shown to be effective and less costly [18]. The proportion of women who sought help from CHWs increased from 5 to 76%. And the proportion of mothers using modern tablets to treat malaria in children almost doubled. Appropriate treatment increased from 3 to 49%. Adequacy of length of treatment increased from 21 to 72%. The average net cost per resident child was US$ 0.06.

#### Economic evaluations of CHW interventions aimed at promoting exclusive breastfeeding

##### Setting

Five studies were conducted from a variety of settings: South Africa [17], Uganda [19], United Kingdom [31], and two from the USA [28, 29]. Of the five studies, two were conducted from a societal perspective [29, 31].

##### Description of intervention

The main purpose of these interventions was to promote exclusive breastfeeding during both the antenatal period and postpartum. In these studies, mothers received individual support at home. The intensity (i.e. the number of visits per mother) of these visits varied substantially. The number of planned visits in the various studies were 3 [28, 29], 5 [19], 10 [31], and 18 [17]. Two studies included a facility-based intervention. In one study, this was in a form of group education for pregnant mothers during a facility antenatal care visit [17, 28]. In the economic evaluation conducted by Frick et al. [28], women received telephonic support in addition to the hospital and home visit support.

##### Results

The cost-effectiveness of the interventions was measured using natural units, in this instance breastfeeding rates [33], months of exclusive breastfeeding (EBF) [17], cost per week of EBF [19], and frequency of illness in infants [29]. A few studies measured intermediate outcomes such as cost per mother [19, 28], per individual counselling visit [19], and per clinic [28].

An economic evaluation of support to mothers through individual counselling by CHW increased EBF prevalence. The cost per mother counselled was US$ 139 and cost per visit was US$ 26. The cost per week of EBF was US$ 15 at 12 weeks postpartum [19].

Postnatal support for women at home showed no significant difference of breastfeeding rates at 6 weeks [33]. The total mean NHS cost to 6-month follow-up for the intervention group was £180 per woman greater than for the control group (£79.60–£272.40, 95% CI).

An intensive intervention of individual breastfeeding support for mothers was cost-effective and also more costly compared to usual care [17]. The cost per supported month of EBF was US$ 41, cost per increased month of EBF was US $48, and the incremental cost-effectiveness ratio was US$ 126.

An intervention comprising of hospital, home visit, and telephone support for 6 months after delivery increased duration in low-income women compared to usual care [29]. At 6 months, 30% of infants were exclusively breastfed (EB) in the intervention arm compared to 15% in usual care. Infants in the intervention group had fewer sick visits. The cost per mother was US$ 301. No incremental cost-effectiveness ratios were calculated.

This intervention conducted in the USA was aimed at increasing breastfeeding rates at 6 weeks and reported reduced medical care utilization and formula feeding costs [28]. The cost per woman was US$ 296. Health care use savings were significant for clinic visits at 4 weeks with the intervention group experiencing 40% less visits.

#### Economic evaluations of CHW interventions aimed at reducing asthma amongst children

##### Setting

In this intervention conducted in the USA, CHWs worked as part of a multidisciplinary team of paediatric pulmonologists, epidemiologists, and intervention coordinators [30]. The study was conducted from a provider perspective.

##### Description of intervention

CHWs did not have prior training on asthma management and were trained for 5 days. As members of the community, they were well acquainted with cultural norms and practice of the target community. The training was conducted by a certified asthma educator and included asthma-related topics, asthma medication and devices, asthma triggers, asthma trigger avoidance and low-cost ways of reducing triggers, and warning signs of an asthma episode and asthma severity. CHWs also received ongoing training.

The CHW conducted four home visits during a 6-month period with participating family and facilitated the establishment of relationship between the family and primary care provider.

##### Results

The efficiency of the asthma control programme was assessed using a cost-savings analysis. Primary outcomes for the intervention included asthma symptoms, asthma-related health resource utilization (HRU) and activity-limited days, and asthma caregiver-related quality of life.

Symptom frequency was reduced by 35%, and urgent health resource utilization was reduced by 75% between the pre- and post-intervention periods. Parental quality of life also improved by a level that is both clinically and statistically significant. The intervention resulted in cost savings; for every US$ 1 spent on intervention, US$ 5.58 is saved. Urgent HRU decreased from median of 4 to 1 (75% decline). Activity-limited days reduced from a median of 7 to 3.5 (50% decline). Regular clinic visits increased from 2.5 to 3.5.

#### Economic evaluations of CHW interventions aimed at reducing malnutrition

##### Setting

Two studies were conducted in Bangladesh [24] and Jamaica [27]. One study was conducted from a provider perspective [27] and the other from a societal perspective [24].

##### Description of interventions

The CHW interventions focussed on providing nutritional advice [24, 27], monitoring growth [24, 27], screening children for malnutrition [24, 27], and treating malnutrition in the community [24] as well as referring malnourished children to the clinics [27]. The studies were not focussed solely on malnutrition, and CHWs had other tasks including counselling communities on nutrition, health, and sanitation. Furthermore, they used algorithms to deliver community case management of basic childhood illness including diarrhoea and acute respiratory infection [24]. Other tasks included following children who had dropped out of immunization programmes [24]. In the economic evaluation conducted by Melville and colleagues [27], CHWs referred children with diarrhoea to clinics. In the study by Puett and colleagues [24], CHWs provided follow-up visits at home to cases of severe acute malnutrition (SAM) without medical complications. Their activities included distributing a weekly ration of Plumpy’Nut (Nutriset, Malaunay, France) and monitoring growth in children.

In both studies, the training of CHWs was similar in terms of intensity and varied in terms of content. CHWs received an initial training of about 1 week. Thereafter, they received monthly refresher training and ongoing supervisory support to implement the community case management of severe acute malnutrition (SAM).

##### Results

One study measured cost-effectiveness using DALYs [24]. The other study was a partial economic evaluation-costing analysis [27]. Both studies were found to be efficient: one cost-effective [24] and the other [27] of moderate cost in its context.

The community case management of SAM in Bangladesh cost US$ 26 per DALY averted, compared with US$ 1344 per DALY averted for inpatient treatment. The average cost to participant households for their child to recover from SAM in community treatment was one-sixth that of inpatient treatment [24]. In the study by Melville et al. [27], malnutrition levels as measured by weight for age decreased by 34.5%. Eighty-one percent of the children gained adequate weight over a 2-year period. The overall cost of the programme per child over the 2-year period was US$ 31.1 (annual cost of US$ 14.50).

#### Economic evaluations of CHW interventions aimed at improving children’s physical health and psychomotor development

##### Settings

Two economic evaluations identified were conducted in Chile in an extremely poor neighbourhood [26] and in Pakistan [21]. Only one study explicitly stated that it was conducted from the provider perspective [21].

##### Description of the intervention

In the economic evaluation by Aracena et al. [26], the intervention was targeted at adolescent single mothers who conceived a first child between 14 and 19 years old. CHWs (referred to as health educators in the study) under supervision of nurse midwives carried out home visits, and there were at least 12 visits, each 1 h in duration, from the third trimester until the child was 1 year old. Standard health care for adolescent single mothers consists of 10 prenatal consultations with nurse midwife [26]. The intervention sought to (1) encourage the young women’s development of their identity as a woman, adolescent, and mother; (2) help her develop life plans; (3) reinforce her parenting skills; (4) promote basic health care practices for both mother and child; and (5) strengthen the adolescent’s relationships with those around her.

In the economic evaluation conducted by Gowani et al. [21], the intervention was delivered using an existing CHW programme. They delivered an integrated responsive stimulation and nutrition intervention to infants to improve early childhood development. CHWs conducted a combination of monthly group and individual home visits. Group visits were approximately 80 min each, and home visits ranged from 7 to 30 min.

##### Results

The efficiency of the two intervention studies above was assessed by a cost-effectiveness analysis [21, 26].

Findings from the Aracena et al. study [26] showed only the following outcomes had a statistically significant difference: (1) development of children’s language skills, (2) nutritional state of the mother, and (3) mental health of the mother. Cost of standard care was US$ 50 per adolescent over a period of 15 months, and median cost of intervention for the home visit programme was US$ 90 per adolescent over a period of 15 months. An investment of US$ 13.50 for a total of 15 months in the home visit programme results in improved mental health of an adolescent mother.

A cost-effectiveness analysis of the results verifies that early childhood interventions that include responsive stimulation are more cost-effective than a nutrition intervention alone in promoting children’s early development. Costs of a responsive stimulation intervention integrated in an existing community-based service providing basic health and nutrition care is approximately US$ 4 per month per child [21].

#### Economic evaluations of CHW interventions aimed at reducing mortality of neonates, children under five, improving maternal health

##### Setting

Three identified economic evaluations were conducted in Nepal [23] and two in Pakistan [20, 22]. All three studies were conducted from a provider’s perspective.

##### Description of intervention

In the economic evaluation conducted by Borghi et al. [23], the intervention was health service strengthening and this was achieved through training government health staff on essential newborn care and provision of basic supplies and equipment. CHWs convened monthly meetings with women’s groups.

In the economic evaluation by Barzgar et al. [20], CHWs delivered primary care and mobilized communities for health. In the economic evaluation by Hafeez et al. [22], CHWs provided education on antenatal care, immunization services, provision of family planning, and basic curative care. Each CHW house was declared a Health House where people can come in for emergencies and to receive basic advice. CHWs were also responsible for record keeping and tracking of health indicators. The economic evaluation conducted in Ecuador [25] was done from the provider-patient perspective. CHWs in each community and spent a total of 10 days on vaccination campaign.

##### Results

The cost-effectiveness of the interventions was measured using generic economic evaluation measures, mortality, and disease- or condition-specific measures and intervention process measures.

The average annual cost per woman of reproductive age was US$ 4.38 (US$ 5.22 with health service strengthening). The average annual cost per newborn infant was US$ 22.51 (US$ 26.82 with health systems strengthening (HSS)). Neonatal deaths averted were US$ 30.94. Cost per neonatal death averted was US$ 5 801 (US$ 6912 with HSS). Life years saved per death averted was 27.54. The cost per life year saved was US$ 211 (US$ 251 with HSS). Total life years saved were 852 [23].

The intervention was aimed at reducing infant, child, and maternal mortality within a year and it generated positive perceptions of family planning. This intervention was found to be cost-effective [20]. A 50% reduction in infant mortality from 130/1000 at baseline to 64/1000 after intervention was observed. An above 50% reduction in maternal mortality, i.e. 596 per 100 000 at baseline to 246 per 100 000 after intervention, was also observed. Another outcome measure that had a more than 50% reduction was infant diarrhoeal deaths. Finally, a more than 97% reduction in neonatal tetanus was observed. There was no observable impact on low birth weight, malnutrition, and pneumonia. Immunization coverage increased by 80, 70, and 100% in Chakwal, Mastung, and Malir, respectively. Costs per person were US$ 0.39 for capital costs and US$ 1.13 per person for recurrent costs.

The national CHW programme was found to be more efficient compared to the status quo. Eighty percent of children were fully immunized under the CHW programme compared to 47% in the areas that are not covered by CHWs. Infant mortality rate was 51% in the CHW-covered areas compared to 39% in non-CHW-covered areas. Maternal mortality rate was 180 compared to 276 in non-CHW-covered areas. Finally, the average cost of each CHW was US$ 570 per year [22].

## Discussion

This review highlights the cost-effectiveness of CHW interventions for health issues that contribute substantially to the ongoing burden of disease in low- and middle-income countries. In addition, this review covers an important topic for human resources for health in particular for health systems in low- and middle-income countries that are trying to scale up interventions to meet various population needs including the emerging non-communicable diseases. There is evidence of cost-effectiveness of CHWs interventions in reducing malaria, asthma, and mortality of neonates and children under 5 years of age. Other economic evaluation studies show evidence of cost-effectiveness in improving exclusive breastfeeding, malnutrition, physical health and psychomotor development in children, and maternal health.

This review is based on all four types of economic evaluation—cost-effectiveness, cost utility, cost benefit, and cost minimization analysis. Studies were not excluded on the basis of scale of implementation (i.e. CHW programmes implemented as vertical programmes or integrated). A systematic approach was used to identify and select economic evaluation studies and synthesizing data across studies and assess the quality of the evidence obtained.

### Limitations

It was not possible to pool the studies and conduct a meta-analysis due to a limited number of identified studies per outcome. Furthermore, the economic evaluations we identified had varied measures of economic evaluation outcomes, for instance disease- or condition-specific outcomes, morbidity, mortality, and generic measures (e.g. disability-adjusted life years (DALY)).

This review focused only on studies published in peer-reviewed journals, in English. Thus, a language as well as a publication bias may have been introduced.

Our findings were consistent with Perry and Zulliger [10] only on cost-effectiveness of community-based therapeutic care for children with severe malnutrition, malaria control programmes, perinatal and neonatal care programmes, and child survival programmes with multiple interventions. It is worth noting that even though our findings are consistent with Perry and Zulliger’ s [10] with respect to certain interventions, our evidence was drawn from a different pool of studies. They identified 33 studies whilst the present study included 19 studies. There was only one common study between these two reviews. Perry and Zulliger’s [10] review differs in how they have selected studies for inclusion. They appear to have selected studies with bundled interventions. It is difficult to know what type of studies their review selected because the methodology does not have sufficient detail. We specifically excluded any studies that had CHWs as one component and for which one could not directly link the impact of the intervention to CHWs.

To aid decision-making on which interventions are cost-effective and should be implemented in a particular context, economic evaluations in this review used different criterion for assessing cost-effectiveness. These included recommendations from a macro-economic commission, the World Bank, and World Health Organization. The WHO Commission on Macroeconomics and WHO-CHOICE threshold guidelines stipulate that an intervention is (1) highly cost-effective if the cost-effectiveness ratio is less than three times gross domestic product per capita, (2) cost-effective if the cost-effectiveness ratio is between one and three times gross domestic product per capita, and (3) not cost-effective if the cost-effectiveness ratio is greater than three time gross domestic product per capita [13]. All interventions were found to be cost-effective or highly cost-effective in their respective countries. The use of these thresholds is methodologically sound in the context of implicit budgets. These thresholds serve as a guide and indicate that a country can potentially invest in that intervention. For instance, the expenditure on health varies between low- and middle-income countries. Thus, these thresholds can be seen as the first level of priority setting. It is crucial for countries to embark on a country-specific priority setting that is informed by their epidemic profile (infant mortality and morbidity), current state of human resources, and availability of human resource.

### Implications for future research

It is somewhat encouraging that there has been an increase in the number of economic evaluations conducted on CHW interventions. However, costs of crucial components of CHW programmes continue to be poorly reported. These include supervision, start-up costs (i.e. intervention development, recruitment, and initial training), and ongoing training costs. Only 7 out of the 19 included studies reported indirect costs, and only 1 study reported start-up costs. It is this level of detail that will inform national programme planning and budgeting.

Ten out of 19 studies reviewed in this paper covered a population of 1000 or more participants. The remaining 9 covered a population size ranging from 41 to 726 participants. Majority of these studies were stand-alone interventions. Thus, future research must focus on economic evaluations of multi-country, integrated CHW programmes. Several countries^7^ have national CHW programmes that vary in coverage of the population, number of CHW trained, and year of initiation of the national programme. These studies can provide insightful lessons on economies of scale and scope that can be achieved or not achieved in CHW programmes operated at a national level.

In the meantime, countries which have decided to initiate or expand their national CHW programmes are faced with the decision of which package of intervention would deliver the best value (i.e. better health outcomes) for their investment. Modelling resource implications for packages of interventions with demonstrated effectiveness using tools like the Lives Saved Tool (*LiST*) (http://www.livessavedtool.org/) and One Health (http://www.who.int/choice/onehealthtool/en/) can aid countries to make better informed decisions in the absence of empirical evidence from primary studies.

Given the importance of CHWs in achieving universal health coverage, it is to be expected that research on effectiveness and cost-effectiveness of CHWs will continue. Thus, there is a need for an update review of economic evaluation of CHW intervention in 5 years’ time. This review must be a systematic review, include both published and non-published material, and it should be broader in scope. Furthermore, the review should address other conceptual issues such as costing community-based interventions versus costing CHW programmes as well as the importance of community participation or engagement.

## Conclusions

The growing body of evidence for cost-effectiveness of community health workers in improving child health outcomes presents an enormous opportunity for governments in low- and middle-income countries to invest in child survival. However, a stronger evidence base on the cost-effectiveness of specific packages of interventions delivered by community health workers is necessary to guide country health policy and programme implementation.

## Additional file

Additional file 1: Search strategy. (DOCX 21 kb)

## Footnotes

1 CMAs in this type of economic evaluation comparisons of interventions are only based on costs, and outcomes are assumed to be identical. CMA seeks to answer the question, what is the least costly way to obtain an outcome?

2 CEA is of most use in situations where a decision marker, operating with a given budget, is considering a limited range of options within a given field. In a CEA, outcomes are measured in their natural units or life years saved.

3 CUA is a form of economic evaluation where the consequences of programmes are adjusted by health state preference scores or utility weights. In general terms, this means that one can assess the quality of life years gained, not just the crude number of years. The most common measure of consequences in CUAs is the quality-adjusted life year.

4 CBA is a form of economic evaluation that values consequences of programmes in money terms, so as to make them commensurate with the costs. This is the broadest form of analysis.

5 CC is a form of economic evaluation where decision-makers are presented with treatments or programmes with multiple objectives along with an array of the differential achievements, along each dimension, for the various alternatives. These data can then be presented to the decision-makers, so that they can make their own trade-off between effects.

6 A costing analysis is a partial economic evaluation.

7 Bangladesh, Thailand, Brazil, Haiti, Ethiopia, Mozambique, Pakistan, and South Africa.
