# Supplementary material for: A systematic review of economic evaluations of CHW interventions aimed at improving child health outcomes
Source: Hum Resour Health. 2017 Feb 28;15:19. doi: 10.1186/s12960-017-0192-5 (PMC5331680; doi:10.1186/s12960-017-0192-5)
Supplement: Additional file 1: — Search strategy. (DOCX 21 kb) [file 12960_2017_192_MOESM1_ESM.docx]

**Additional file 1**

Search Name: Child health interventions

Last Saved: 12/05/2014 12:27:55.637

Description:

ID Search

#1 MeSH descriptor: [Pregnancy Outcome] explode all trees

#2 cost-benefit analysis

#3 (lay or voluntary or volunteer* or untrained or unlicensed or nonprofessional* or non next professional*) near/5 (worker* or visitor* or attendant* or aide or

aides or support* or person* or helper* or carer* or caregiver* or care next giver* or consultant* or assistant* or staff or visit* or midwife or midwives):ti or (lay or voluntary

or volunteer* or untrained or unlicensed or nonprofessional* or non next professional*) near/5 (worker* or visitor* or attendant* or aide or aides or support* or person* or

helper* or carer* or caregiver* or care next giver* or consultant* or assistant* or staff or visit* or midwife or midwives):ab or lay next volunteer*:ti or lay next volunteer*:ab

#4 "pregnancy":ti,ab,kw and "cost-benefit analyses" (Word variations have been searched)

#5 #4 and #3

#6 infant mortality in Economic Evaluations

#7 #3 and #6

#8 (trained near/3 (volunteer* or (health next worker*) or mother*)):ti or (trained near/3 (volunteer* or (health next worker*) or mother*)):ab in Economic Evaluations

#9 (community or village*) near/3 ((health next worker*) or (health next care next worker*) or (healthcare next worker*)):ti or (community or village*) near/3 ((health

next worker*) or (health next care next worker*) or (healthcare next worker*)):ab in Economic Evaluations

#10 #6 and #9

#11 community health workers in Economic Evaluations

#14 "community health worker":ti,ab,kw and infant:ti,ab,kw in Economic Evaluations (Word variations have been searched)

#15 #1 and #3 in Economic Evaluations

#16 lay health workers in Economic Evaluations

#17 "peer support":ti,ab,kw in Economic Evaluations (Word variations have been searched)

#18 "untrained personnel":ti,ab,kw in Economic Evaluations (Word variations have been searched)

#19 "untrained personnel":ti,ab,kw in Economic Evaluations (Word variations have been searched)

#20 "pregnancies":ti,ab,kw (Word variations have been searched)

#21 #20 and #11

#22 "child birth":ti,ab,kw (Word variations have been searched)

#23 #22 and #11

#24 #1 and #11

#25 "child birth":ti,ab,kw (Word variations have been searched)

| [# ▲](http://ovidsp.tx.ovid.com/sp-3.12.0b/ovidweb.cgi?&S=JNPAFPDEEADDOIGLNCMKOFMCDCGJAA00&Sort+Sets=descending) | **Searches** | **Results** | **Search Type** |
| --- | --- | --- | --- |
| 1 | (lay support and cost-effectiveness and pregnanc*).tw. | 5 | Advanced |
| 2 | economic evaluation.ab. or economic evaluation.kw. or economic evaluation.tw. | 8619 | Advanced |
| 3 | (lay support* or peer support* or village health worker).ab. | 665 | Advanced |
| 4 | (pregnanc* or child birth or infant mortalit*).ab. | 56313 | Advanced |
| 5 | (cost effectiveness or cost or cost benefit).ab. | 60039 | Advanced |
| 6 | (cost effectiveness or cost or cost benefit).tw. | 529106 | Advanced |
| 7 | 3 and 4 and 5 | 0 | Advanced |
| 8 | 3 and 4 and 6 | 5 | Advanced |
| 9 | community health worker?.tw. | 2919 | Advanced |
| 10 | community health worker?.ab. | 333 | Advanced |
| 11 | 4 and 6 and 9 | 52 | Advanced |
| 12 | 2 and 6 and 9 | 24 | Advanced |
| 13 | traditional birth*.ab. | 95 | Advanced |
| 14 | 5 and 13 | 11 | Advanced |
| 15 | volunteer health worker.ab. | 0 | Advanced |
| 16 | midwife.ab. | 703 | Advanced |
| 17 | 2 and 16 | 5 | Advanced |
| 18 | vitamin A.ab. | 20420 | Advanced |
| 19 | breastfeed*.ab. | 3551 | Advanced |
| 20 | hand wash*.ab. | 385 | Advanced |
| 21 | (diarrhea or diarrhoea).ab. | 12021 | Advanced |
| 22 | 6 and 9 and 21 | 36 | Advanced |
| 23 | 6 and 9 and 18 | 19 | Advanced |
| 24 | 4 and 6 and 20 | 2 | Advanced |
| 25 | 5 and 9 and 19 | 2 | Advanced |
| **26** | **2 and 10 and 19** | **0** | **Advanced** |

**PEDE Search criterion:**

**Keywords:** (TITLE_ABSTRACT_KEYWORDS "community(-| )health(-| )worker")

**Age groups:** Perinates Neonates Infants Children Adolescents

**Years:** 1980 - 2014

**Keywords:** (TITLE_ABSTRACT_KEYWORDS "volunteer")

**Age groups:** Perinates Neonates Infants Children Adolescents

**Years:** 1980 - 2014

| [# ▲](http://ovidsp.tx.ovid.com/sp-3.12.0b/ovidweb.cgi?&S=JNPAFPDEEADDOIGLNCMKOFMCDCGJAA00&Sort+Sets=descending) | **Searches** | **Results** | **Search Type** |
| --- | --- | --- | --- |
| 1 | (lay support and cost-effectiveness and pregnanc*).tw. | 5 | Advanced |
| 2 | economic evaluation.ab. or economic evaluation.kw. or economic evaluation.tw. | 8619 | Advanced |
| 3 | (lay support* or peer support* or village health worker).ab. | 665 | Advanced |
| 4 | (pregnanc* or child birth or infant mortalit*).ab. | 56313 | Advanced |
| 5 | (cost effectiveness or cost or cost benefit).ab. | 60039 | Advanced |
| 6 | (cost effectiveness or cost or cost benefit).tw. | 529106 | Advanced |
| 7 | 3 and 4 and 5 | 0 | Advanced |
| 8 | 3 and 4 and 6 | 5 | Advanced |
| 9 | community health worker?.tw. | 2919 | Advanced |
| 10 | community health worker?.ab. | 333 | Advanced |
| 11 | 4 and 6 and 9 | 52 | Advanced |
| 12 | 2 and 6 and 9 | 24 | Advanced |
| 13 | traditional birth*.ab. | 95 | Advanced |
| 14 | 5 and 13 | 11 | Advanced |
| 15 | volunteer health worker.ab. | 0 | Advanced |
| 16 | midwife.ab. | 703 | Advanced |
| 17 | 2 and 16 | 5 | Advanced |
| 18 | vitamin A.ab. | 20420 | Advanced |
| 19 | breastfeed*.ab. | 3551 | Advanced |
| 20 | hand wash*.ab. | 385 | Advanced |
| 21 | (diarrhea or diarrhoea).ab. | 12021 | Advanced |
| 22 | 6 and 9 and 21 | 36 | Advanced |
| 23 | 6 and 9 and 18 | 19 | Advanced |
| 24 | 4 and 6 and 20 | 2 | Advanced |
| 25 | 5 and 9 and 19 | 2 | Advanced |
| **26** | **2 and 10 and 19** | **0** | **Advanced** |

**PEDE Search criterion:**

**Keywords:** (TITLE_ABSTRACT_KEYWORDS "community(-| )health(-| )worker")

**Age groups:** Perinates Neonates Infants Children Adolescents

**Years:** 1980 - 2014

**Keywords:** (TITLE_ABSTRACT_KEYWORDS "volunteer")

**Age groups:** Perinates Neonates Infants Children Adolescents

**Years:** 1980 - 2014
